# Data for serum 1,5 anhydroglucitol concentration in different populations

**DOI:** 10.1016/j.dib.2018.08.165

**Published:** 2018-09-01

**Authors:** Marciane Welter, Kátia C. Boritza, Mauren I. Anghebem-Oliveira, Railson Henneberg, Aline B. Hauser, Fabiane G.M. Rego, Geraldo Picheth

**Affiliations:** a Post Graduate Program in Pharmaceutical Sciences, Federal University of Parana, Curitiba, Parana, Brazil; b Department of Clinical Analysis, Federal University of Parana, Rua Prefeito Lothário Meissner, 632, 80210-170 Curitiba, Parana, Brazil

## Abstract

1,5 anhydroglucitol (1,5-AG), is a nonmetabolized 1-deoxy form of glucose, originate mainly from the diet. 1,5-AG is a biomarker to detect and magnify hyperglycemic excursions (postprandial hyperglycemia) in diabetic patients. Concentrations of 1,5-AG has been applied as supporting biomarker to diagnosis of the major forms of diabetes (type 1, type 2, and gestational). The serum 1,5-AG reference interval is relevant to the appropriate clinical application of this biomarker. This article contains data regards to serum concentration of the biomarker primarily for healthy subjects, capture from the literature, in different populations. Correlation analysis between 1,5-AG and markers associated with diabetes and its complication were presented. The data was complementary to the study “Reference intervals for serum 1,5-anhydroglucitol in children, adolescents, adults, and pregnant women” (Welter et al., 2018). The data present in this article improve the comparisons for 1,5-AG in different conditions and methodologies.

**Specifications Table**

**Table:** 

Subject area	Clinical laboratory
More specific subject area	*Biomarkers for clinical chemistry associated with diabetes*
Type of data	*Tables*
How data was acquired	*Capture from literature data for 1,5-AG concentration and methodology. Comparison reference interval data was obtained with enzymatic colorimetric assay (Glycomark) measured in automate system (LabMax 400, Labtest, Brazil)*
Data format	*Analyzed*
Experimental factors	*Blood samples (serum or plasma EDTA) measured with different methodologies*
Experimental features	*Compilation, calculations, analysis (descriptive statistics) and comparison of literature data*
Data source location	*Federal University of Parana, Curitiba, Brazil.*
Data accessibility	*Data is in published papers and in this article.*
Related research article	M. Welter, K.C. Boritza, M.I. Anghebem-Oliveira, R. Henneberg, A.B. Hauser, F.G.M. Rego, G. Picheth, Reference intervals for serum 1,5-anhydroglucitol in children, adolescents, adults, and pregnant women, CCA (2018) [1].

**Value of the data**

• Data will facilitate the comparison of 1,5-AG in different studies.
• The data showed the correlation among 1,5-AG and relevant parameters associated with diabetes.
• The data provide comparison between 1,5-AG reference interval in different ethnicities, ages, gender and methodologies.
• These data provide information to researchers and clinical laboratory professionals to improve the 1,5-AG diagnostic use.

## 1 Data

In this article, we provide complementary data (urea, total protein and lipid profile) of the studied groups (Table 1), correlation analysis (Table 2) and comparisons from the literature for serum 1,5-anhydroglycitol (1,5-AG) concentration, to our study. We proposed a reference interval (Tables 3 and 4) for this biomarker in children, adolescent, adults and pregnant women [2].

**Table 1:** Complementary laboratory characteristic of studied groups.

**Parameters**	**Children n = 580**	**Adolescents n = 496**	**Adults n = 922**	**Pregnant women n = 305**
Sex (M/F)	242/338	192/304	460/462	–
Urea, mmol/L	3.8 (3.1–4.5)	3.8 (3.2–4.3)	4.3 (3.5–5.3)	3.4 (2.8–4.3)
Total Protein, g/L	81 (74–89)	78 (71–85)	71 (67–74)	69 (64–74)
Cholesterol, mmol/L	4.1 (3.6–4.6)	3.9 (3.4–4.4)	4.4 (4.0–5.4)	5.2 (4.1–6.2)
HDL-c, mmol/L	1.4 (1.2–1.6)	1.3 (1.0–1.5)	1.4 (1.1–1.5)	1.2 (1.0–1.6)
LDL-c, mmol/L	2.1 (1.7–2.5)	2.1 (1.7–2.6)	2.5 (2.0–3.1)	3.0 (2.5–4.1)
Triglycerides, mmol/L	1.1 (0.8–1.5)	0.8 (0.6–1.1)	1.4 (1.0–2.0)	1.3 (1.0–2.0)

Values are median (25–75%; interquartile range); M, male and F, female

HDL-c, high density lipoprotein-cholesterol; LDL-c, low density lipoprotein-cholesterol

Abbreviations: BMI, Body mass index; n, sample size.

**Table 2:** Significative (*P* < 0.05) Spearman rank order correlation of 1,5 anhydroglucitol with glycemia, HbA1c, age, body mass index (BMI) and creatinine.

**Groups**	**Sex**	**1,5-AG correlation (R)**				
		**Glycemia**	**HbA1c**	**Age**	**BMI**	**Creatinine**
Children (0–14 y)	Male	NS	NS	0.133	NS	NS
	Female	NS	0.128	NS	NS	0.163
Adolescents (14–18 y)	Male	NS	0.221	0.153	0.151	NS
	Female	NS	NS	0.221	NS	NS
Adults (≥18 y)	Male	NS	NS	−0.144	NS	NS
	Female	NS	NS	−0.102	NS	NS
Combining all		NS	NS	−0.310	−0.187	0.112
Pregnant women	**Gestation weeks**					
n = 110	<23 weeks	NS	–	NS	NS	NS
n = 106	24–28 weeks	NS	–	NS	NS	NS
n = 52	29–32 weeks	NS	–	NS	NS	0.402
n = 37	>32	NS	–	NS	NS	NS
Combining pregnant		0.095	–	−0.155	NS	NS

NS, non-significant; –, data no available.

Y, years old.

**Table 3:** Serum 1,5-anhydroglucitol reference intervals and concentrations in different healthy populations.

	**1,5-AG, µmol/L**					**Studies/Methodology**
**Sex**	**Male**			**Female**		
**Subjects**	**R.I.**		**n**	**R.I.**	**n**	
Children and adolescents (5–18 years)	(92–298)		432	(84–278)	642	Our study [2] Enzymatic colorimetric GlycoMark^TM^
US adolescents (12–18 years)	(95–178)		6	(140–172)	5	[3] Enzymatic colorimetric GlycoMark^TM^
	[88–212]			[120–180]		
	150 ± 31			150 ± 15		
	(95–178)					
	[102–198]					
	150 ± 24					
US young	158 ± 40		82	143 ± 37	54	[4] Enzymatic colorimetric GlycoMark^TM^
				(54–227)		
(10–29 years)	(63–271)			[69–217]		
	[78–238]					
	Total (*n* = 136) 151 ± 39 (54–271); [73–229]					
	(<18 years)158 ± 35 [88–228]					
	(>18 years) 137 ± 42 [53–221]					
Adults (19–79 years)	(80–260)		460	(62–241)	462	Our study [2] Enzymatic colorimetric GlycoMark^TM^
Finland adults (25–50 years)	93 mean		29	77 mean	110	[5] Gas chromatography (GC)
	81 mean; 10–146 range (*n* = 139)					
US adults (18–39 years)	(61–207)		224	(37–195)	224	[6] Enzymatic colorimetric GlycoMark^TM^
US adults (18–39 years)	(52−178)		875	(50−166)	924	[7]
Australian adults (40 ± 13 years)	125 ± 41 (*n* = 95)					[8] Enzymatic colorimetric GlycoMark^TM^
	[43–207]					
German adults	157 ± 44 (*n* = 116)					[9] Liquid chromatography–mass Spectrometric (LC–MS)
	[69–245]					
Chinese Mauritians	144 ± 51 (*n *= 82)					[10] Enzymatic pyranose oxidase
	[42–246]					
	(98–195)					
Chinese adults (22–80 years)	182 ± 39			159 ± 52		[11] Enzymatic pyranose oxidase
	[104–260]			[55–263]		
	92–294 (*n *= 57)					
Chinese adults >20 and <40 years >50 years	176 ± 46 [84–268]		82	116 ± 35 [46–186]	185	[12] Liquid chromatography negative ion electrospray tandem mass spectrometry (LC–MS/MS)
	166 ± 67 [32–300]		9	122 ± 41 [40–204]	14	
Chinese adults	(83.1–240.7)					[13] Enzymatic
	161.9 ± 40.2					
	[81.5–242.3] (*n *= 120)					
Chinese adults (22–78 years)	190 ± 54		254	160 ± 49	322	[14] Enzymatic colorimetric GlycoMark^TM^
	[82–298]			[62–258]		
	(69–278)					
	[67–279]					
	173 ± 53					
Chinese adults (20–79 years)	(107–367)		226	(79–306)	232	[15] Enzymatic Medical system, Ningbo, China
	226.3 ± 60.7			175.2 ± 55.8		
	[104.9–347.7]			[63.6–286.8]		
Japanese (18–81 years)	132 ± 36 (*n* = 45)					[16] Gas–liquid chromatography (GLC)
	[60–204]					
Japanese (mean 47 years)	145 ± 44 (*n *= 229)					[17] Gas–liquid chromatography (GLC)
	[57–233]					
Japanese (27–68 years)	(114–215)					[18] Gas–liquid chromatography (GLC)
	158 ± 38					
	[82–234]					
Japanese adults (23–76 years)	159.8 ± 9.8 (*n* = 20)					[19] Enzymatic Nippon–Kayaku
	[140–179]					
Japanese adults (30–79 years)	140 ± 56		991	122 ± 43	1104	[20] Enzymatic Kyowa Medex Co.
	[28–252]			[36–208]		
Japanese adults	137.1 ± 8.2		181	120.6 ± 39	203	[21] Enzymatic Lana Nippon–Kayaku
	[120.7–153.5]		231	[42.6–198.6]	519	
	(50.5 ± 9.7 y)			(54.1 ± 10.3)		
	124.3 ± 45.1			115.1 ± 40.2		
	[34.1–214.5]			[34.7–195.5]		
	(74.5 ± 5.8 y)			(75.3 ± 6.7 y)		

US, Americans from United States; UK, English; n, sample size.

Values were reference interval (2.5th–97.5th); [95% calculated as mean±2–SD]; mean±SD or median (IQR, interquartile range, 25–75%).

**Table 4:** Serum 1,5-anhydroglucitol reference intervals and concentrations in pregnancy in different populations.

**Pregnant**	**1,5-AG, μmol/L R.I.**	**n**	**Studies/Methodology**
Pregnant Women <23 weeks of gestation	(56–298)	110	Our study [2] Enzymatic colorimetric GlycoMark^TM^
Pregnant Women >24 weeks of gestation	(33–181)	195	Our study [2] Enzymatic colorimetric GlycoMark^TM^
Japanese healthy non-pregnant women	113 ± 32	25	[22] Gas-liquid chromatography (GLC)
	[49–197]		
Japan pregnant women at 36 weeks gestation	62 ± 28	543	
	[6–118]		
Japan women on 5th day of puerperium	66 ± 23	543	
	[20–112]		
Japan women on 30th day of puerperium	87 ± 21	543	
	[45–129]		
Japan pregnant Women at >24 weeks of gestation	128 (IQR 102–160)		
UK Normoglycemic women with glycosuric pregnancy (~31 weeks)	46	16	[23] High-performance liquid chromatography (HPLC)
	(IQR 30–56)		
UK Normoglycemic women without glucosuric pregnancy (~31 weeks)	72	16	
	(IQR 55–79)		
UK Normoglycemic women (~31 weeks)	55 (IQR 31–72)	32	
Chinese pregnant women (16–45 years) at 26–28 weeks of gestation	133.0 ± 52.9	44	[24] Enzymatic pyranose oxidase
	[27.2–238.8]		

US, Americans from United States; UK, English; n, sample size.

Values were reference interval (2.5th–97.5th); [95% calculated as mean±2−SD]; mean±SD or median (IQR, interquartile range, 25–75%).

We studied healthy Euro-Brazilian subjects, classified as children (0–14 years old), adolescent (>14 and <18 years old) and adults (≥18 years old). Additionally, we analyzed pregnant women in four gestational periods, <23 weeks; 24–28 weeks, 29–32 weeks and >32 weeks of gestation. 1,5-AG was measure by enzymatic colorimetric method (Glycomark^TM^; Tomen America, New York, NY, USA) in automated system Labmax 400 analyzer (Labtest Diagnostic).

The laboratory parameters, markers for kidney function (urea), nourishment (total protein) and lipid profile were compatible with healthy subjects (Table 1).

The correlation in healthy subjects between 1,5-AG and glycemia, HbA1c, age, BMI and creatinine were weak or none (Table 2).

## 2 Experimental design, materials and methods

### 2.1 Study population

The population comprises 2303 unrelated Euro-Brazilian healthy subjects from Curitiba, State of Parana, South of Brazil [1]. All samples were obtained with the approval of the Ethics Committee of the Federal University of Parana.

Adult samples (*n* = 922) were collected from blood bank donors. Children (*n* = 580) and adolescent samples (*n* = 496) were obtained from Public Schools. Healthy pregnant women (*n* = 305) samples were obtained from the Curitiba Government Laboratory.

The normoglycemic criteria applied for selected subjects in the study were fasting glycemia <5.5 mmol/L with an HbA1c range of 20.2–36.6 mmol/mol (4.0–5.7%) for children, adolescents, and adults. For pregnant women a fasting blood glucose <5.1 mmol/L was applied to exclude gestational diabetes.

All subjects declared that were not using any medications or drugs.

### 2.2 Samples

Samples were serum obtained in non-fasting state for adults, children and adolescents, and fasting for those who were pregnant. Blood were collected in BD vacutainers SST II advance vacutainer with silica clot activator/gel (Becton Dickinson Co.). Bloods were separated in less than two hours from venipuncture and the serum stored in an ultrafreezer (−80 °C).

### 2.3 Analytical methods

Concentrations of 1,5-AG were measured enzymatically with the Glycomark reagent (GlycoMark, Tomen America, New York, NY Inc.) in an automated system (Labmax 400 analyzer; Labtest. Diagnostics). The reaction details and methodology performance were described in Nowatzkea et al. [6].

### 2.4 Clinical and laboratory parameters

Clinical data acquisition and analytical procedures, for laboratory data, have been reported previously [2].

### 2.5 Data analysis

Descriptive statistics, correlation analysis and reference intervals were calculated with MedCalc MedCalc version 17.6 (MedCalc Statistical Software bvba, Ostend, Belgium). Probability values (*p*-values) less than 5% (*p* < 0.05) were considered significant for all tests.
